# Influence of Age on Associations of Occlusal Status and Number of Present Teeth with Dementia in Community-Dwelling Older People in Japan: Cross-Sectional Study

**DOI:** 10.3390/ijerph20095695

**Published:** 2023-05-01

**Authors:** Hikaru Shiraki, Satoko Kakuta, Ji-Woo Park, Taishi Aosa, Toshihiro Ansai

**Affiliations:** 1 Division of Community Oral Health Development, Kyushu Dental University, 2-6-1 Manazuru, Kokurakita-ku, Kitakyushu 803-8580, Japan; r19shiraki@fa.kyu-dent.ac.jp (H.S.); r15kakuta@fa.kyu-dent.ac.jp (S.K.); ppp3366@hanmail.net (J.-W.P.); 2 Department of Food and Nutrition, Beppu University, Beppu 874-8501, Japan; aosa@nm.beppu-u.ac.jp

**Keywords:** oral health, dementia, functional tooth units, tooth loss

## Abstract

While occlusal status has been reported to be related to cognitive function, little is known about the influence of age on that relationship. The present study examined the associations of tooth loss and occlusal status with dementia in the older people, as well as the effects of age on those relationships. A total of 196 older participants (median age: 84 years) were enrolled. Occlusal status was assessed using functional tooth units (FTU), calculated based on the number of paired natural or artificial teeth. Logistic regression analysis was then performed using dementia as the objective variable, and FTU or number of teeth as explanatory variables. The results showed that higher FTU was associated with lower risk of dementia. Furthermore, when stratified by median age, the association was greater for those aged less than 84 years. On the other hand, there was no significant association of number of present teeth with dementia. These results suggest that the risk of dementia is lower for individuals with better occlusion and that occlusal factor may have a greater effect on dementia onset in younger older people. It is thus recommended that both occlusal function and age be incorporated as factors in programs developed for dementia prevention.

## 1. Introduction

The number of people with dementia worldwide is projected to reach 152 million by 2050 [1], while the global societal economic cost of dementia by 2030 is estimated to increase up to USD 2 trillion [2]. Furthermore, the number of people with dementia in Japan is expected to increase to 7 million by 2025 [3]. According to a survey by the Japanese Ministry of Health, Labor and Welfare, dementia is the most common cause of long-term care need [4]. Therefore, early detection and control of risk factors for dementia are important components of a preventive policy to reduce socio-economic costs and extend healthy life expectancy. 

Dementia is a multifactorial disease, with modifiable risk factors, such as lower level of education, hypertension, hearing impairment, smoking, obesity, depression, physical inactivity, diabetes, low level of social contact, excessive alcohol consumption, traumatic brain injury, and air pollution, identified as related [5]. Furthermore, a systematic review presented findings showing a link between oral health and cognitive function, and also noted associations of learning memory, complex attention, and executive function with oral health [6]. Other studies have reported a putative link between tooth loss and cognitive impairment/dementia [7,8], with the former suggesting that such an association can be explained by a pathway through poor mastication caused by tooth loss. In addition, in a more recent investigation, a putative link between posterior occlusion and cognitive function was discovered [9]. However, few studies have assessed differences related to the association of dementia with occlusal status or number of present teeth, though masticatory performance and occlusal factors are known to be closely related. 

Age has been reported to have an effect on the associations of dementia with its risk factors [5]. For example, a recent large-scale study that used data from the Swedish Twin Registry reported that the risk effect of cardiometabolic disease on dementia was attenuated when the disease developed in a late life stage [10]. However, to the best of our knowledge, no study has investigated the influence of age on the association of oral functional status, including occlusal status and number of present teeth, and dementia. 

Several preventive programs for dementia have been proposed, such as reduction in risk of cognitive decline and dementia in the WHO guidelines proposed by the WHO [11] and the Framework for Promoting Dementia Care presented by the Japanese government [4]. On the other hand, to date, no preventive policy that incorporates influence of age on associations of oral-related risk factor and dementia in older people has been proposed.

Using those findings, we hypothesized that occlusal status has a greater influence on dementia in older people as compared to number of present teeth and that influence is greater in younger individuals. Our aim was to examine the associations of occlusal status and number of present teeth with dementia in community-dwelling older adults in Japan stratified by age.

## 2. Materials and Methods

### 2.1. Study Population

**Figure 1:**
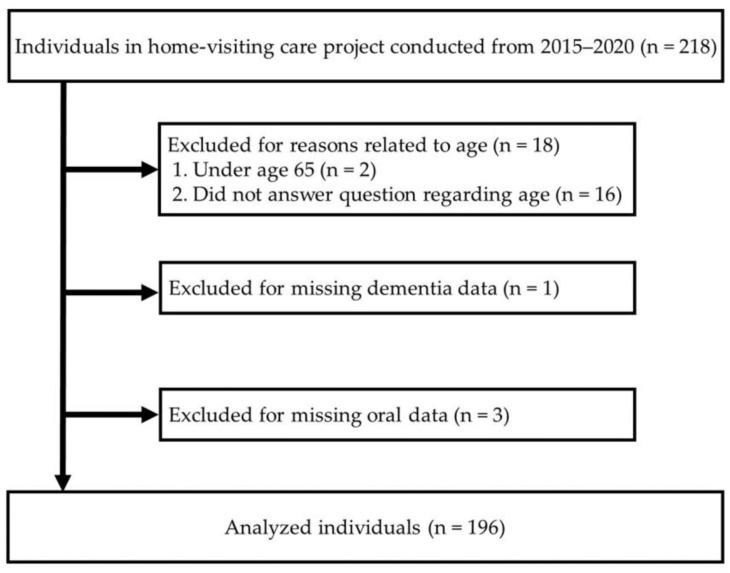
Flow diagram of participant selection.

This cross-sectional study was conducted using data obtained from the Home-Visiting Oral Care Project, which was implemented in Buzen City, Fukuoka Prefecture, from April 2015 to November 2020. The city is located in the western part of Kyushu and has a population of about 25,000, with most primarily engaged in agriculture and fishing. Eligibility criteria for this project including the following: (1) resident of Buzen city, (2) aged 40 years or more, and (3) covered by the National Health Insurance and Late-stage Senior Citizens Health Insurance systems of Japan. As a result, a total of 218 citizens were initially enrolled. Of those, participants aged <65 years (n = 2), with missing age (n = 16), dementia data (n = 1), and oral data (n = 3) were excluded, for a total of 196 (females 64.3%, males 35.7%, age range 65 to 97 years) included in the present analyses (Figure 1). This study was conducted after gaining approval from the ethics committee of our university (no. 20–31). Details were explained in writing and orally, and written informed consent from the participants or their guardians was obtained prior to beginning the study.

### 2.2. Assessment of Dental Status

Dental status including number of present teeth and artificial teeth was determined by a trained dentist prior to the study [12]. Occlusal status was assessed using functional tooth units (FTU) defined as number of natural teeth or fixed/removable dental prosthesis counterparts. Exclusion criteria based on a method previously reported [13] with some modifications were as follows: third molars present, teeth with mobility of 3, and ill-fitting denture teeth, since dental problems such as mobile teeth or an ill-fitting denture are known to cause chewing difficulty. The FTU scores ranged from 0 to 14, with a higher score considered to represent better occlusal condition.

### 2.3. Dementia Assessment

Information for determining dementia was obtained from a report provided by the primary care physician of the participant and based on the National Health Insurance database. Dementia or cognitive decline was clinically diagnosed by either a psychiatrist or neurologist working in the local community.

### 2.4. Nutritional Status Assessment

Nutritional status was assessed using body mass index (BMI) and the Mini Nutritional Assessment^®^-Short-Form (MNA^®^-SF) [14]. BMI was calculated by measuring height and weight, and dichotomized based on the presence of obesity (BMI ≥ 30). The MNA^®^-SF was distributed as a questionnaire to the participant or their guardian, and the possible score ranged from 0 to 14, with higher scores representing better nourishment, and then dichotomized based on well-nourished status (MNA^®^-SF ≥ 12) for this study.

### 2.5. Medical History Assessment

For medical history, data related to hypertension, cardiovascular disease, diabetes mellitus, cerebrovascular disease, and depression were obtained from a report provided by the primary care physician and the National Health Insurance database.

### 2.6. Others

Data for age, gender, education, smoking history, and drinking habits were obtained using a questionnaire distributed to the participant or their guardian.

### 2.7. Statistical Analysis

All analyses were performed using R (version 4.1.2) with a significance level of 5%. The characteristics of the participants were analyzed according to dementia status; then, subgroup analysis after stratification by age was performed. A Mann–Whitney U test was used for continuous variables or discrete variables, and Fisher’s exact test for categorical variables. Logistic regression analysis was used to test the hypotheses, with FTU or number of present teeth as the explanatory variable and dementia as the objective variable. Covariates entered into the model were selected based on previous studies [5,7]. In addition, subgroup analysis stratified by age was performed, as age difference has been reported to have an effect on the association between cognitive function and tooth loss [15]. FTU, number of present teeth, and MNA^®^-SF were treated as discrete variables. When multicollinearity was present, *a priori* inclusion of covariates in the logistic regression model was decided. A variance inflation factor cutoff value of 4.0 was used to assess multicollinearity, though no variables exhibit multicollinearity in the present model.

## 3. Results

### 3.1. Characteristics of Study Population according to Dementia Status

The number of participants with dementia was 47 (24.0%) and the number of those without dementia was 149 (76.0%). Table 1 shows the study population characteristics according to dementia status. Median age was 84.0 years (IQR: 77.75, 88.00); 64.3% were female and 35.7% male. The dementia group showed associations with older age (*p* < 0.001), hypertension (*p* = 0.018), depression (*p* < 0.001), lower MNA^®^-SF (*p* = 0.012), lower FTU (*p* = 0.008), and smoking history (*p* = 0.025) compared to the non-dementia group. No significant associations were determined for the other variables between the groups.

### 3.2. Characteristics of Study Population according to Dementia Status—Subgroup Analysis Stratified by Age

Table 2 shows characteristics of the study population according to dementia status stratified by age. Among those aged ≥84 years (n = 99), the number of patients with dementia was 34 (34.3%) and the number of those without dementia was 65 (65.7%). The dementia group was found to be associated with hypertension (*p* = 0.011) and depression (*p* = 0.038) in this age group compared to the non-dementia group. Among those aged <84 years (n = 97), 13 (13.4%) were with and 84 (86.6%) without dementia. The dementia group showed associations with depression (*p* = 0.017) and lower FTU (*p* = 0.023) compared to the non-dementia group, while no significant associations were detected for the other variables.

### 3.3. Associations of FTU and Number of Present Teeth with Dementia

Results of logistic regression analyses for the association of dementia, FTU, and number of present teeth are presented in Table 3. Three models included FTU and were adjusted for age, gender, and education (Model 1: odds ratio (OR) 0.88, 95% confidence interval (CI) 0.81–0.97, *p* = 0.007), hypertension, diabetes mellitus, and smoking history (Model 2: OR, 0.86, 95% CI, 0.78–0.95, *p* = 0.003), or MNA^®^-SF (Model 3: OR, 0.87, 95% CI, 0.78–0.96, *p* = 0.006). The results showed dementia to be significantly associated with lower FTU. The three models including number of present teeth were adjusted for age, gender, and education (Model 1: OR, 1.00, 95% CI, 0.97–1.04, *p* = 0.864), hypertension, diabetes mellitus, and smoking history (Model 2: OR, 1.00, 95% CI, 0.96–1.04, *p* = 0.980), or MNA^®^-SF (Model 3: OR, 1.01, 95% CI, 0.97–1.05, *p* = 0.707). Those results indicated that dementia was not significantly associated with number of present teeth.

### 3.4. Associations of Dementia and FTU—Subgroup Analysis Stratified by Age

Results of logistic regression analyses for the associations of dementia and FTU stratified by age are shown in Table 4. The median age (84 years) of the participants was used as the cut-off value for subgroup analysis. For those aged ≥84 years, three models including FTU were adjusted for age, gender, and education (Model 1: OR, 0.91, 95% CI, 0.82–1.01, *p* = 0.086), hypertension, diabetes mellitus, and smoking history (Model 2: OR, 0.86, 95% CI, 0.75–0.98, *p* = 0.026), or MNA^®^-SF (Model 3: OR, 0.89, 95% CI, 0.77–1.00, *p* = 0.064). The results indicated that dementia was not significantly associated with FTU in participants aged ≥84 years. On the other hand, for those <84 years old, significant association was determined using the three models (Model 1: OR, 0.81, 95% CI 0.68–0.96, *p* = 0.015; Model 2: OR, 0.82, 95% CI, 0.68–0.96, *p* = 0.016; Model 3: OR, 0.82, 95% CI, 0.68–0.97, *p* = 0.018).

### 3.5. Associations of Dementia, and Number of Present Teeth—Subgroup Analysis Stratified by Age

Results of logistic regression analyses for the associations of dementia and number of present teeth stratified by age are shown in Table 5. In the participants aged ≥84 years, number of present teeth was not determined to have a significant association in any of the models (Model 1: OR, 1.00, 95% CI, 0.95–1.05, *p* = 0.941; Model 2: OR, 0.99, 95% CI, 0.94–1.04, *p* = 0.764; Model 3: OR, 1.01, 95% CI, 0.95–1.06, *p* = 0.843). Furthermore, for those <84 years old, there was no significant association indicated by any of the models (Model 1: OR, 1.02, 95% CI, 0.95–1.10, *p* = 0.608; Model 2: OR, 1.01, 95% CI, 0.94–1.10, *p* = 0.738; Model 3: OR, 1.01, 95% CI, 0.94–1.09, *p* = 0.783).

### 3.6. Influence of Age on Association of Dementia, FTU, and Number of Present Teeth

**Figure 2:**
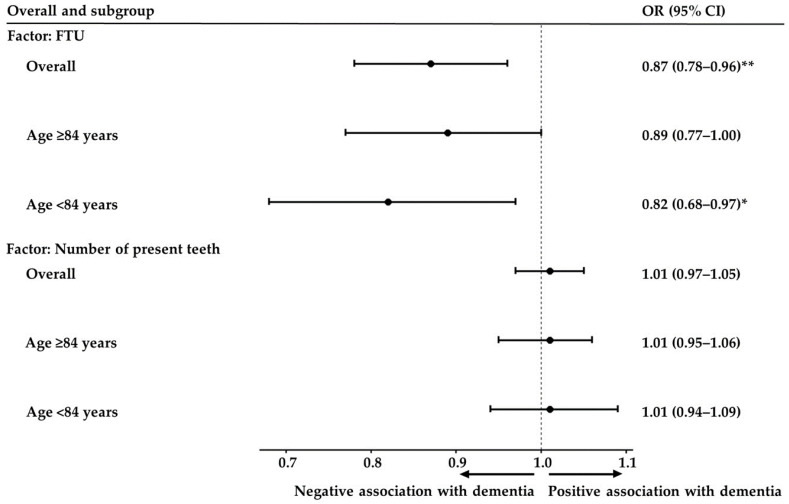
Logistic regression analysis of associations of dementia with FTU and number of present teeth in all participants, as well as those aged ≥84 years and <84 years. FTU, Functional Tooth Unit; OR, odds ratio; CI, confidence interval. Results shown are based on Model 3, with adjustments for age, gender, education, hypertension, diabetes, smoking history, and Mini Nutritional Assessment^®^-Short Form. Points and horizontal lines show values for OR (95% CI) and vertical dashed lines indicate odds ratio = 1. * *p* < 0.05 and ** *p* < 0.01.

Logistic regression analysis results for the association of dementia with FTU and number of present teeth in all participants and those aged ≥84 and <84 years are presented in Figure 2. Age of <84 years showed a greater effect (OR, 0.82, 95% CI, 0.68–0.97, *p* = 0.018) compared to the results for all participants (OR, 0.87, 95% CI, 0.78–0.96, *p* = 0.006) on the association between dementia and FTU. In contrast, age of ≥84 years did not demonstrate a significant association. Furthermore, dementia was not significantly associated with number of present teeth using any of the models.

## 4. Discussion

Findings of the present cross-sectional study of community-dwelling adults aged ≥65 years old indicate a decreased OR for the presence of dementia in older people with good occlusal status and a stronger influence of occlusal factors in younger people (<84 years). On the other hand, there was no association between dementia and number of present teeth. 

An association of dementia with occlusal status has been noted in other reports [16,17]. In a matched case-control study, there was a significant reduction in occlusal teeth contact in the dementia group noted during follow-up examinations [16]. Furthermore, Popovac et al. [17] suggested that a greater number of occlusal contacts was a more important protective factor for Alzheimer’s disease than having more teeth, whereas whether the functional contacts originated from natural teeth or dentures did not affect the outcomes in that study.

On the other hand, Dintica et al. [18] discovered that there was no significant association between a pattern of posterior occlusion with dementia in the Swedish Adoption/Twin Study of Aging. 

However, several investigations, including those presented by Ko et al. [16], Popovac et al. [17] and Dintica et al. [18], did not assess status of anterior teeth, mobility teeth, or denture fitting, which may be related to the different outcomes noted in the present compared to those of previous studies. To the best of our knowledge, the present investigation is the first to evaluate relationships with dementia using comprehensive occlusal indices including anterior teeth, tooth mobility teeth, and denture fitting. 

Recently, evidence has been reported regarding the relationship of tooth loss with cognitive function. A systematic review reported by Cerutti-Kopplin et al. [7] showed that tooth loss was associated with cognitive decline. In contrast, there was no association between number of present teeth and dementia in the present study. Interestingly, Lexomboon et al. [19] suggested that there is no association between cognitive function and tooth loss, and that the difference between natural teeth and a prosthesis may not contribute significantly to cognitive impairment as long as the individual has no chewing difficulty. Similarly, Takeuchi et al. [9] reported no association between cognitive function and tooth loss, which is consistent with the present results. Other studies used various means for analysis of tooth number, such as a cut-off value of 20 teeth [7], self-reported number of teeth [19], or three divisions (1–9, 10–19, ≥20) [9]. Discrete values were used for assessment of number of teeth in the present study. 

Livingstone et al. [5] reported findings showing that level of education, hypertension, hearing impairment, smoking, obesity, depression, physical inactivity, diabetes, social contact, excessive alcohol consumption, traumatic brain injury, and exposure to air pollution are risk factors for dementia. In the overall final adjusted model of the present study, hypertension was significantly associated with dementia, while other factors, including education, smoking history, and diabetes mellitus, were not (Table 3). Depression and obesity could not be included in the model because the number of cases was extremely small (Table 1). In addition, no data were available related to hearing impairment, physical inactivity, low level of social contact, traumatic brain injury, air pollution exposure, or excessive alcohol consumption. Therefore, findings obtained in this study may not fully capture the complex relationship between oral health and dementia.

Inflammation has been implicated to be involved in the mechanism of dementia onset. Arrivé et al. [20] pointed out that tooth loss may reduce the risk of dementia by suppression of chronic inflammation caused by periodontal disease. The differences between those and the present findings might be due to variations in measurement methods and pathophysiological conditions of chronic inflammation. Nevertheless, the relationship between number of teeth and cognitive function may require careful consideration.

Few studies have presented findings indicating that the association between dementia and oral-related risk factors is influenced by age. Tsakos et al. [15] demonstrated that edentulous condition was an independent predictor of slower walking speed and worse memory in subjects aged 60 to 74 years, but not in those aged 75 and older in a prospective study of community residents aged 60 and older in the United Kingdom. Another prospective study of subjects aged 45 to 64 years (mean 63 years) conducted in the United States demonstrated that periodontal disease and edentulism were associated with increased risk of dementia and mild cognitive impairment, with that association stronger in younger (≤62 years) subjects [21]. However, neither of these studies used occlusal indexes for their analyses. The present findings demonstrated an influence of age on the association between dementia and FTU, indicating that incorporation of age factors may lead to development of effective dementia prevention programs. However, the sample size for the subgroup analyses might not have been sufficient to draw strong conclusions and the results should be interpreted with caution. Additional studies with larger sample sizes will be needed to confirm these findings.

An association between dementia and occlusal status appears to be biologically plausible. Mastication is known to be associated with occlusal status and number of present teeth [22], with masticatory function decreasing in association with tooth loss and decreased occlusal pairs. Findings obtained in animal and epidemiologic studies have suggested that loss of occlusion has effects on cognitive function [23,24]. The putative pathway is reduced masticatory stimulation that leads to decreased cerebral blood flow, inactivation of the cortical area, and lower blood oxygen level [25,26]. In the present study, differences in the association of dementia with occlusal status between individuals less than 84 and 84 years or older were detected. Roher et al. [27] also reported that blood flow to the brain decreases with age. Thus, even when good masticatory function is present, cognitive function may decline due to decreased cerebral blood flow with aging, though the association of cerebral blood flow with mastication was not investigated in this study.

Another mechanism has been proposed. Since poor nutritional status is associated with FTU and number of present teeth [28], it is possible that tooth loss and loss of occlusion lead to poor nutritional status. Nutrition-related factors have been indicated as potentially contributing to dementia. For example, it was reported that walnut consumption improves the balance between free radicals and antioxidants, thus attenuating amyloid beta protein (Aβ)-induced oxidative stress and associated Aβ-mediated cell death [29]. However, the present results obtained with the MNA^®^-SF indicated no significant association between dementia and nutritional status, which may have been due to the greater influence of occlusion factors compared to nutrition-related factors.

The WHO [11] has proposed guidelines to reduce the risk of cognitive decline and dementia, though oral-related risk factors are not mentioned. In Japan, while the importance of management of oral function is described in the Framework for Promoting Dementia Care [3], no specific strategy is included. At the municipal level, some measures including number of times chewing during meals have been proposed in guidelines for prevention of dementia. Such approaches are quite limited at the present time. 

This study has several limitations. First, because the present study had a cross-sectional design, causal relationship between occlusal status and dementia cannot be established. Second, generalizability of the findings is considered to be difficult due to the study population being limited to residents of Buzen City. Third, data from local health and welfare program agencies were used, thus a preliminary sample size calculation was not performed. Finally, no data related to socioeconomic factors, such as social contact and income, were obtained, though education was included in the analytical model. A longitudinal cohort study will be needed to verify the present results.

## 5. Conclusions

The present study discovered an association of loss of occlusion with dementia in older people, with the effect of occlusion greater in the younger participants. Moreover, age was shown to have a crucial role in those associations. Therefore, when constructing a program for prevention of dementia, incorporation of age into the program as well as maintenance of occlusal condition may be helpful for devising an effective strategy.

## Figures and Tables

**Table 1:** Characteristics of study population according to dementia status (n = 196).

		Overall		
Variable		Non-Dementia	Dementia	
		(n = 149)	(n = 47)	*p* *
Age, years		82 (76.0, 87.0)	88 (82.5, 90.5)	<0.001
Gender	Female	91 (61.1)	35 (74.5)	0.117
	Male	58 (38.9)	12 (25.5)	
Hypertension	No	88 (59.1)	18 (38.3)	0.018
	Yes	61 (40.9)	29 (61.7)	
Cardiovascular disease	No	111 (74.5)	37 (78.7)	0.698
	Yes	38 (25.5)	10 (21.3)	
Diabetes mellitus	No	118 (79.2)	40 (85.1)	0.526
	Yes	31 (20.8)	7 (14.9)	
Cerebrovascular disease	No	138 (92.6)	40 (85.1)	0.147
	Yes	11 (7.4)	7 (14.9)	
Depression	No	149 (100)	42 (89.4)	<0.001
	Yes	0 (0.0)	5 (10.6)	
Cancer	No	129 (86.6)	43 (91.5)	0.370
	Yes	20 (13.4)	4 (8.5)	
BMI, kg/m^2^		22.6 (20.3, 25.2)	21.9 (19.0, 24.1)	0.135
	<30	144 (97.3)	46 (100.0)	0.574
	≥30	4 (2.7)	0 (0.0)	
MNA^®^-SF		12 (11.0, 13.0)	11 (9.0, 12.0)	0.012
	<12	62 (41.6)	28 (59.6)	0.043
	≥12	87 (58.4)	19 (40.4)	
Number of present teeth		18 (8.0, 24.0)	15 (5.5, 22.5)	0.130
FTU		14 (12.0, 14.0)	12 (8.5, 14.0)	0.008
Denture	No	57 (38.3)	20 (42.6)	0.611
	Yes	92 (61.7)	27 (57.4)	
Denture fitting (upper)	No	15 (18.8)	4 (15.4)	1.000
	Yes	65 (81.2)	22 (84.6)	
Denture fitting (lower)	No	15 (19.0)	7 (33.3)	0.234
	Yes	64 (81.0)	14 (66.7)	
High school graduation or above	No	58 (38.9)	18 (38.3)	1.000
	Yes	91 (61.1)	29 (61.7)	
Smoking experience	No	101 (67.8)	40 (85.1)	0.025
	Yes	48 (32.2)	7 (14.9)	
Drinking habit	No	110 (73.8)	40 (85.1)	0.120
	Yes	39 (26.2)	7 (14.9)	

BMI, body mass index; MNA^®^-SF, Mini Nutritional Assessment^®^-Short Form; FTU, Functional Tooth Unit. Data are shown as number (%) or median (25th, 75th percentile), unless otherwise noted. * Fisher’s exact test for categorical variables and Mann–Whitney U test for continuous or discrete variables.

**Table 2:** Characteristics of study population according to dementia status—subgroup analysis stratified by age (n = 196).

		Age ≥ 84 Years			Age < 84 Years		
Variable		Non-Dementia	Dementia		Non-Dementia	Dementia	
		(n = 65)	(n = 34)	*p* *	(n = 84)	(n = 13)	*p* *
Age, years		87 (85.0, 91.0)	89 (87.0, 91.8)	0.101	77 (71.0, 80.0)	78 (76.0, 82.0)	0.111
Gender	Female	45 (69.2)	25 (73.5)	0.817	46 (54.8)	10 (76.9)	0.227
	Male	20 (30.8)	9 (26.5)		38 (45.2)	3 (23.1)	
Hypertension	No	38 (58.5)	10 (29.4)	0.011	50 (59.5)	8 (61.5)	1.000
	Yes	27 (41.5)	24 (70.6)		34 (40.5)	5 (38.5)	
Cardiovascular disease	No	47 (72.3)	27 (79.4)	0.477	64 (76.2)	10 (76.9)	1.000
	Yes	18 (27.7)	7 (20.6)		20 (23.8)	3 (23.1)	
Diabetes mellitus	No	51 (78.5)	30 (88.2)	0.282	67 (79.8)	10 (76.9)	0.728
	Yes	14 (21.5)	4 (11.8)		17 (20.2)	3 (23.1)	
Cerebrovascular disease	No	59 (90.8)	29 (85.3)	0.504	79 (94.0)	11 (84.6)	0.236
	Yes	6 (9.2)	5 (14.7)		5 (6.0)	2 (15.4)	
Depression	No	65 (100.0)	31 (91.2)	0.038	84 (100.0)	11 (84.6)	0.017
	Yes	0 (0.0)	3 (8.8)		0 (0.0)	2 (15.4)	
Cancer	No	54 (83.1)	31 (91.2)	0.272	75 (89.3)	12 (92.3)	0.739
	Yes	11 (16.9)	3 (8.8)		9 (10.7)	1 (7.7)	
BMI, kg/m^2^		22.3 (19.8, 25.1)	21.5 (18.7, 24.0)	0.377	22.9 (20.8, 25.4)	22.2 (20.6, 24.1)	0.397
	<30	62 (96.9)	33 (100)	0.546	82 (97.6)	13 (100.0)	1.000
	≥30	2 (3.1)	0 (0.0)		2 (2.4)	0 (0.0)	
MNA^®^-SF		12 (10.0, 13.0)	11 (9.0, 12.0)	0.116	12 (11.0, 13.3)	12 (10.0, 12.0)	0.133
	<12	32 (49.2)	22 (64.7)	0.202	30 (35.7)	6 (46.2)	0.543
	≥12	33 (50.8)	12 (35.3)		54 (64.3)	7 (53.8)	
Number of present teeth		11 (1.0, 21.0)	11 (2.0, 19.8)	0.985	22.5 (13.0, 26.0)	21 (12.0, 26.0)	0.695
FTU		14 (11.0, 14.0)	13 (10.3, 14.0)	0.079	14 (12.0, 14.0)	12 (8.0, 13.0)	0.023
Denture	No	17 (26.2)	12 (35.3)	0.361	40 (47.6)	8 (61.5)	0.387
	Yes	48 (73.8)	22 (64.7)		44 (52.4)	5 (38.5)	
Denture fitting (upper)	No	9 (19.6)	3 (14.3)	0.740	6 (17.6)	1 (20.0)	1.000
	Yes	37 (80.4)	18 (85.7)		28 (82.4)	4 (80.0)	
Denture fitting (lower)	No	11 (25.6)	6 (33.3)	0.547	4 (11.1)	1 (33.3)	0.345
	Yes	32 (74.4)	12 (66.7)		32 (88.9)	2 (66.7)	
High school graduation or above	No	38 (58.5)	14 (41.2)	0.138	20 (23.8)	4 (30.8)	0.730
	Yes	27 (41.5)	20 (58.8)		64 (76.2)	9 (69.2)	
Smoking experience	No	49 (75.4)	29 (85.3)	0.308	52 (61.9)	11 (84.6)	0.131
	Yes	16 (24.6)	5 (14.7)		32 (38.1)	2 (15.4)	
Drinking habit	No	54 (83.1)	28 (82.4)	1.000	56 (66.7)	12 (92.3)	0.100
	Yes	11 (16.9)	6 (17.6)		28 (33.3)	1 (7.7)	

BMI, body mass index; MNA^®^-SF, Mini Nutritional Assessment^®^-Short Form; FTU, Functional Tooth Unit. Data are shown as number (%) or median (25th, 75th percentile), unless otherwise noted. * Fisher’s exact test for categorical variables and Mann–Whitney U test for continuous or discrete variables.

**Table 3:** Logistic regression models for associations of FTU and number of present teeth with dementia.

**Factor: FTU**								
**Variable**	**Crude Model**		**Model 1**		**Model 2**		**Model 3**	
	**OR (95% CI)**	***p***	**OR (95% CI)**	***p***	**OR (95% CI)**	***p***	**OR (95% CI)**	***p***
FTU (per one-point increase)	0.89 (0.82–0.97)	0.006	0.88 (0.81–0.97)	0.007	0.86 (0.78–0.95)	0.003	0.87 (0.78–0.96)	0.006
Age, years (per one-point increase)			1.12 (1.06–1.19)	<0.001	1.11 (1.05–1.19)	<0.001	1.11 (1.05–1.18)	<0.001
Male (ref: Female)			0.61 (0.27–1.32)	0.222	1.29 (0.46–3.49)	0.620	1.30 (0.46–3.56)	0.615
High school graduation or above Yes			1.71 (0.81–3.76)	0.167	1.83 (0.83–4.16)	0.140	1.70 (0.77–3.89)	0.196
Hypertension Yes					2.47 (1.15–5.49)	0.023	2.86 (1.29–6.61)	0.011
Diabetes mellitus Yes					0.75 (0.26–1.95)	0.568	0.76 (0.26–2.02)	0.597
Smoking experience Yes					0.32 (0.09–1.03)	0.062	0.33 (0.09–1.06)	0.069
MNA^®^-SF (per one-point increase)							0.86 (0.73–1.01)	0.069
**Factor: Number of Present Teeth**								
**Variable**	**Crude Model**		**Model 1**		**Model 2**		**Model 3**	
	**OR (95% CI)**	***p***	**OR (95% CI)**	***p***	**OR (95% CI)**	***p***	**OR (95% CI)**	***p***
Number of present teeth (per one-point increase)	0.98 (0.94–1.01)	0.152	1.00 (0.97–1.04)	0.864	1.00 (0.96–1.04)	0.980	1.01 (0.97–1.05)	0.707
Age, years (per one-point increase)			1.12 (1.06–1.18)	<0.001	1.11 (1.05–1.18)	<0.001	1.10 (1.04–1.18)	<0.001
Male (ref: Female)			0.60 (0.27–1.28)	0.200	1.10 (0.40–2.92)	0.847	1.08 (0.39–2.91)	0.878
High school graduation or above Yes			1.75 (0.84–3.78)	0.141	1.94 (0.90–4.32)	0.096	1.79 (0.83–4.01)	0.147
Hypertension Yes					2.05 (1.00–4.31)	0.053	2.46 (1.16–5.41)	0.021
Diabetes mellitus Yes					0.65 (0.23–1.67)	0.394	0.69 (0.24–1.80)	0.461
Smoking experience Yes					0.38 (0.12–1.17)	0.101	0.40 (0.12–1.23)	0.118
MNA^®^-SF (per one-point increase)							0.83 (0.71–0.98)	0.023

OR, odds ratio; CI, confidence interval; FTU, Functional Tooth Unit; MNA^®^-SF, Mini Nutritional Assessment^®^-Short Form. Model 1; adjusted for gender, age, high school graduation or above. Model 2; additionally adjusted for hypertension, diabetes mellitus, smoking experience. Model 3; additionally adjusted for MNA^®^-SF.

**Table 4:** Logistic regression models for associations of FTU and dementia—subgroup analysis stratified by age.

**Subgroup: Age ≥ 84 Years**								
**Variable**	**Crude Model**		**Model 1**		**Model 2**		**Model 3**	
	**OR (95% CI)**	***p***	**OR (95% CI)**	***p***	**OR (95% CI)**	***p***	**OR (95% CI)**	***p***
FTU (per one-point increase)	0.91 (0.82–1.01)	0.079	0.91 (0.82–1.01)	0.086	0.86 (0.75–0.98)	0.026	0.89 (0.77–1.00)	0.064
Age, years (per one-point increase)			1.10 (0.98–1.26)	0.116	1.09 (0.95–1.26)	0.205	1.10 (0.96–1.28)	0.178
Male (ref: Female)			0.84 (0.31–2.20)	0.733	2.27 (0.58–9.43)	0.243	2.44 (0.62–10.28)	0.207
High school graduation or above Yes			2.11 (0.89–5.18)	0.096	2.70 (1.01–7.67)	0.053	2.53 (0.93–7.35)	0.076
Hypertension Yes					5.32 (1.92–16.57)	0.002	6.48 (2.23–21.71)	0.001
Diabetes mellitus Yes					0.35 (0.08–1.36)	0.149	0.35 (0.07–1.36)	0.147
Smoking experience Yes					0.27 (0.05–1.27)	0.109	0.26 (0.05–1.27)	0.105
MNA^®^-SF (per one-point increase)							0.83 (0.67–1.02)	0.078
**Subgroup: Age < 84 Years**								
**Variable**	**Crude Model**		**Model 1**		**Model 2**		**Model 3**	
	**OR (95% CI)**	***p***	**OR (95% CI)**	***p***	**OR (95% CI)**	***p***	**OR (95% CI)**	***p***
FTU (per one-point increase)	0.85 (0.74–0.99)	0.035	0.81 (0.68–0.96)	0.015	0.82 (0.68–0.96)	0.016	0.82 (0.68–0.97)	0.018
Age, years (per one-point increase)			1.18 (1.02–1.43)	0.052	1.18 (1.01–1.43)	0.057	1.17 (1.01–1.42)	0.063
Male (ref: Female)			0.30 (0.06–1.16)	0.104	0.48 (0.07–2.70)	0.428	0.46 (0.06–2.67)	0.413
High school graduation or above Yes			1.00 (0.25–4.54)	0.997	1.08 (0.26–5.09)	0.919	1.05 (0.25–4.95)	0.948
Hypertension Yes					0.74 (0.18–2.80)	0.658	0.85 (0.20–3.43)	0.815
Diabetes mellitus Yes					1.29 (0.23–5.88)	0.750	1.43 (0.25–6.73)	0.661
Smoking experience Yes					0.38 (0.04–2.49)	0.342	0.38 (0.04–2.61)	0.360
MNA^®^-SF (per one-point increase)							0.89 (0.66–1.20)	0.414

OR, odds ratio; CI, confidence interval; FTU, Functional Tooth Unit; MNA^®^-SF, Mini Nutritional Assessment^®^-Short Form. Model 1; adjusted for gender, age, high school graduation or above. Model 2; additionally adjusted for hypertension, diabetes mellitus, smoking experience. Model 3; additionally adjusted for MNA^®^-SF.

**Table 5:** Logistic regression models for associations of number of present teeth and dementia—subgroup analysis stratified by age.

**Subgroup: Age ≥ 84 Years**								
**Variable**	**Crude Model**		**Model 1**		**Model 2**		**Model 3**	
	**OR (95% CI)**	***p***	**OR (95% CI)**	***p***	**OR (95% CI)**	***p***	**OR (95% CI)**	***p***
Number of present teeth (per one-point increase)	1.00 (0.96–1.05)	0.991	1.00 (0.95–1.05)	0.941	0.99 (0.94–1.04)	0.764	1.01 (0.95–1.06)	0.843
Age, years (per one-point increase)			1.10 (0.97–1.24)	0.132	1.08 (0.95–1.24)	0.251	1.09 (0.95–1.25)	0.231
Male (ref: Female)			0.83 (0.31–2.16)	0.713	1.91 (0.49–7.71)	0.351	1.98 (0.51–8.12)	0.329
High school graduation or above Yes			2.18 (0.93–5.28)	0.077	2.80 (1.08–7.75)	0.038	2.60 (0.97–7.43)	0.062
Hypertension Yes					4.13 (1.60–11.58)	0.005	5.38 (1.95–16.57)	0.002
Diabetes mellitus Yes					0.31 (0.07–1.15)	0.097	0.31 (0.07–1.19)	0.104
Smoking experience Yes					0.34 (0.06–1.58)	0.185	0.33 (0.06–1.52)	0.168
MNA^®^-SF (per one-point increase)							0.80 (0.65–0.98)	0.030
**Subgroup: Age < 84 Years**								
**Variable**	**Crude Model**		**Model 1**		**Model 2**		**Model 3**	
	**OR (95% CI)**	***p***	**OR (95% CI)**	***p***	**OR (95% CI)**	***p***	**OR (95% CI)**	***p***
Number of present teeth (per one-point increase)	0.99 (0.93–1.06)	0.746	1.02 (0.95–1.10)	0.608	1.01 (0.94–1.10)	0.738	1.01 (0.94–1.09)	0.783
Age, years (per one-point increase)			1.15 (1.00–1.36)	0.080	1.15 (1.00–1.36)	0.077	1.14 (0.99–1.36)	0.085
Male (ref: Female)			0.33 (0.07–1.19)	0.115	0.52 (0.08–2.61)	0.446	0.51 (0.08–2.62)	0.443
High school graduation or above Yes			1.01 (0.27–4.45)	0.989	1.09 (0.28–5.02)	0.903	1.05 (0.27–4.74)	0.945
Hypertension Yes					0.73 (0.19–2.55)	0.631	0.82 (0.21–2.96)	0.758
Diabetes mellitus Yes					1.21 (0.23–5.17)	0.807	1.37 (0.25–6.08)	0.688
Smoking experience Yes					0.39 (0.05–2.42)	0.339	0.40 (0.04–2.49)	0.350
MNA^®^-SF (per one-point increase)							0.88 (0.67–1.18)	0.352

OR, odds ratio; CI, confidence interval; MNA^®^-SF, Mini Nutritional Assessment^®^-Short Form. Model 1; adjusted for gender, age, high school graduation or above. Model 2; additionally adjusted for hypertension, diabetes mellitus, smoking experience. Model 3; additionally adjusted for MNA^®^-SF.

## Data Availability

The datasets analyzed during the current study are available from the corresponding author upon reasonable request.
